# Prevalence of chronic conditions and influenza vaccination coverage rates in Germany: Results of a health insurance claims data analysis

**DOI:** 10.1111/irv.13054

**Published:** 2022-10-01

**Authors:** Oliver Damm, Anya Krefft, Jonas Ahlers, Rolf Kramer, Julian Witte, Manuel Batram, Jörg Schelling, Wolfgang Greiner

**Affiliations:** ^1^ Sanofi‐Aventis Deutschland GmbH Berlin Germany; ^2^ School of Public Health Bielefeld University Bielefeld Germany; ^3^ Vandage GmbH Bielefeld Germany; ^4^ Medical Faculty Ludwig‐Maximilians‐University Munich Germany

**Keywords:** chronic disease, Germany, influenza, human, prevalence, vaccination coverage

## Abstract

**Background:**

The significant annual burden caused by seasonal influenza has led to global calls for increased influenza vaccination coverage rates (VCRs). We aimed to estimate the proportion of the German population at high risk of serious illness from influenza due to chronic conditions and to estimate age‐specific VCRs of people with/without chronic conditions.

**Methods:**

Using health insurance claims data covering nine influenza seasons (2010–2019), we assessed up to 7 million insured individuals per season across all German regions. Individuals were classified according to age and presence of chronic health conditions. VCRs were estimated using outpatient healthcare utilization documentation.

**Results:**

In the 2018–2019 influenza season, 47.3% of individuals had ≥1 chronic condition. Most common were circulatory disorders, accounting for more than a third of individuals with ≥1 condition. Prevalence of chronic diseases, and therefore the proportion of high‐risk individuals, increased slightly over time across most age groups.

A downward trend in influenza VCRs was observed in all age groups until the 2017–2018 season, followed by a noticeable increase in the 2018–2019 season. Highest VCRs occurred among individuals of ≥60 years, with a 38.5% VCR for this age group in the 2018–2019 season. Several factors, including age, chronic condition type, and geographical location, affected VCRs.

**Conclusions:**

Influenza VCRs in individuals at high risk of severe complications from influenza infection are insufficient. Our results suggest that intensified public health efforts are necessary to reach the World Health Organization vaccination coverage target of 75%.

## 1 INTRODUCTION

Each year, influenza epidemics cause considerable morbidity and mortality worldwide. In the 2017–2018 season, the number of deaths attributable to influenza in Europe was estimated to be 152,000.^1^ A study on communicable diseases in Europe revealed that influenza accounts for 30% of the total burden of 31 selected infectious diseases in terms of disability‐adjusted life years (DALYs).^2^ Globally, influenza has also been shown to have a significant economic burden, with both direct and indirect costs. For example, a recent study covering data from 2015 in the United States, across all age groups, estimated that direct medical costs amounted to approximately $3.2 billion nationally.^3^ The same study showed that influenza has a substantial impact on workplace activity, with 20 million days of productivity lost in the United States during 2015.^3^

In 2003, the World Health Organization (WHO) stated that member states should aim to achieve an influenza vaccination coverage rate (VCR) of 75% or higher among older people (defined as those aged ≥65 years in 76% of the responding countries but refers to those aged ≥60 years in Germany) and individuals with chronic illnesses and that this target should be achieved by 2010.^4^ This motion was reaffirmed by the European Parliament and extended in a 2009 European Council recommendation, whereby European countries should reach 75% vaccination coverage in older age groups by 2015.^5^

In Germany, the current influenza immunization recommendation issued by the Standing Committee on Vaccination (STIKO) focuses on individuals aged ≥60 years, individuals with underlying chronic conditions, pregnant women, and healthcare workers.^6^ It is especially important to vaccinate those with chronic conditions because vaccination has been associated with improved clinical outcomes among this population, particularly individuals with chronic respiratory disease and cardiovascular disease.^7^, ^8^ Currently, available information on influenza VCRs for Germany is limited since published evidence does not always include a detailed breakdown by age and/or risk status. Furthermore, large parts of the official VCR surveillance by the Robert Koch Institute (RKI) are only reported graphically. In this study, we first aimed to estimate the proportion of the German population that is at high risk of developing severe influenza complications due to underlying chronic conditions. Second, we aimed to estimate age‐specific influenza VCRs in the overall population and in individuals with and without chronic conditions.

## 2 METHODS

### 2.1 Data source and study period

Our study is based on claims data of a large German Statutory Health Insurance (SHI) fund (BARMER) covering nine consecutive influenza seasons (2010–2011 to 2018–2019). Of the 8.8 million individuals covered by this fund, up to 7 million insured individuals were included in the analysis depending on the season, corresponding to between 6.6% and 8.7% of the national population (supporting information Table S1). Data were included across all regions of Germany. The health insurance claims data contained information on outpatient and inpatient diagnoses and corresponding healthcare resource use.

An influenza season was defined to reach from the beginning of the third quarter of an index year to the end of the second quarter of the following year. This expanded definition of a season was chosen to capture all influenza vaccine administrations throughout a year.

The study period of every influenza season comprises eight quarters, divided into an analysis period of four quarters, preceded by a pre‐observation period of four quarters. For example, the study period for the 2017–2018 season starts in the third quarter of 2016 and ends in the second quarter of 2018 (supporting information Figure S1). The analysis period was used to estimate the VCRs, and the pre‐observation period was used to identify individuals with chronic conditions. For every influenza season, we included all insured individuals who had continuous insurance coverage in the previous season (pre‐observation period) and also had continuous insurance coverage, or died, during the season of interest (analysis period).

As the study did not gather primary patient‐ or individual‐level data or involve any interventions, formal ethical approval was not sought.

### 2.2 Identification of individuals with chronic conditions

For every season, insured individuals with chronic conditions were identified using the International Classification of Diseases (ICD) 10 diagnoses (see Table 1). ICD‐10 code selection was based on previous studies that estimated the size of the population at risk because of chronic conditions^9^, ^10^; codes were added or excluded to reflect the relevant spectrum of chronic conditions.

**TABLE 1:** Chronic conditions and corresponding ICD‐10 codes

Type of chronic condition	ICD‐10 codes
Respiratory	J40‐J47, J96.1, J96.9, E84
Asthma	J45‐J46
COPD	J44
Circulatory	I05‐I09, I10‐I15, I20‐I25, I26‐I28, I30‐I52, I60‐I69, G45, Q20‐Q24, Z99.0‐Z99.1, Z99.4
Hypertension	I10‐I15
Heart failure	I50
Ischemic heart diseases	I20‐I25
Pulmonary heart diseases	I26‐I28
Cerebrovascular diseases	I60‐I69, G45
Diabetes	E10‐E14
Liver	K70‐K77, B18‐B19
Renal	N18‐N19, Z49, Z99.2
Neurological	G20‐G21, G30, G35, G40‐G41, G10, G12.2, F00
Multiple sclerosis	G35
Immunocompromised	C00‐C97 (excluding C44), B20‐B24, D70‐D71, D80‐D90, M05‐M06, M08, M30‐M35, Z51.0, Z51.1, Z94
Cancer	C00‐C97 (excluding C44)
HIV/AIDS	B20‐B24
Disorders involving the immune mechanism	D80‐D90

Abbreviations: AIDS, acquired immunodeficiency syndrome; COPD, chronic obstructive pulmonary disease; HIV, human immunodeficiency virus; ICD, International Classification of Diseases.

Individuals were considered chronically ill if they fulfilled ≥1 of the following criteria in the pre‐observation period: a primary inpatient diagnosis, two same confirmed outpatient diagnoses in two different quarters, two same secondary inpatient diagnoses in two different quarters, or a confirmed outpatient diagnosis and the same secondary inpatient diagnosis in two different quarters. Outpatient diagnoses that were only documented as suspected diagnoses by the respective physician were excluded.

### 2.3 Vaccination status

Analysis of influenza vaccination coverage was based on documentation in the outpatient healthcare utilization database in the season of interest. Age‐specific VCRs (%) were estimated for all insured individuals and those with and without chronic conditions. For the ≥60 years age group, we also estimated the vaccination coverage in the Association of Statutory Health Insurance Physicians (ASHIP) regions.

## 3 RESULTS

### 3.1 Proportion of population with chronic health conditions

**FIGURE 1:**
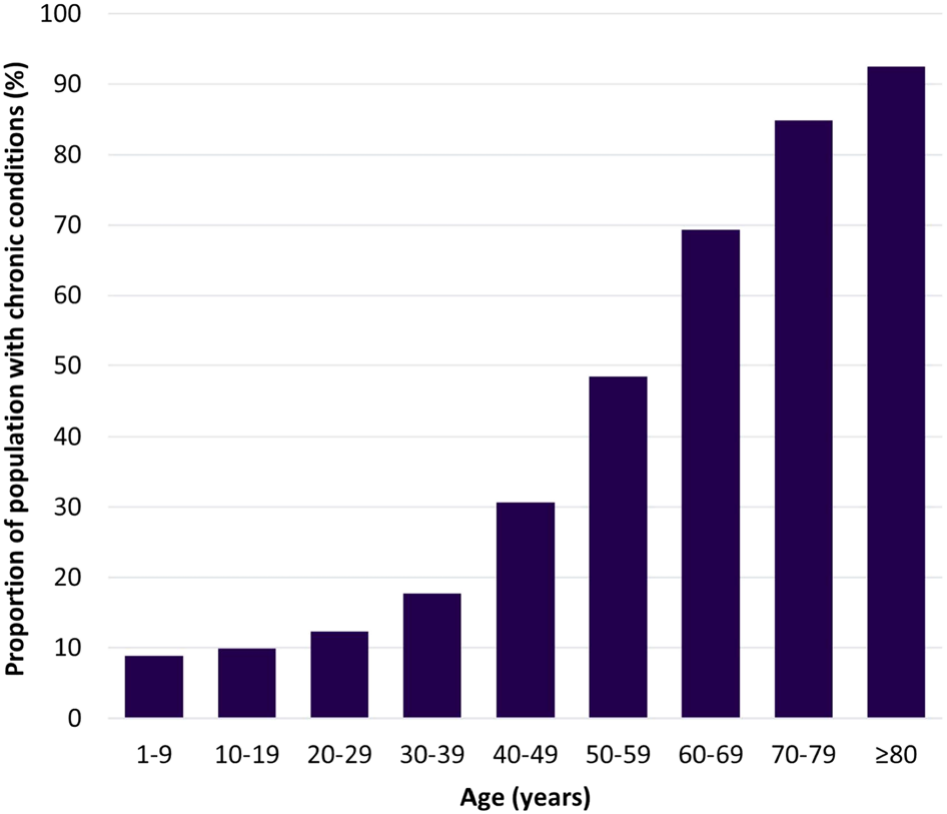
Age‐specific proportion of population with at least one chronic condition in the 2018–2019 season

In the 2018–2019 season (the most recent with data available for our study), 47.3% of the studied population had at least one chronic condition, and age‐specific prevalence ranged from 8.8% in the youngest age group (1–9 years) to 92.4% in the oldest age group (≥80 years) (Figure 1). A similar increase with age was observed in previous seasons (Table 2). Within most age groups, the prevalence of chronic diseases also increased over time.

**TABLE 2:** Age‐specific proportion of population (%) with at least one chronic condition in the 2010–2011 to 2018–2019 seasons

	Age (years)										
Season	1–9	10–19	20–29	30–39	40–49	50–59	60–69	70–79	≥80	≥60	Total
2010–2011	11.4	10.5	11.0	16.1	26.4	45.3	68.1	82.5	90.5	78.2	43.0
2011–2012	11.2	10.3	11.2	16.3	27.2	46.0	68.1	82.9	91.0	78.6	43.7
2012–2013	10.9	10.1	11.3	16.4	28.2	46.7	68.4	83.4	91.5	79.0	44.4
2013–2014	10.4	10.2	11.7	16.8	29.0	47.2	68.6	83.7	91.7	79.3	44.9
2014–2015	10.7	10.2	11.9	17.1	29.5	47.6	69.2	84.3	92.2	80.0	45.8
2015–2016	10.8	10.4	12.4	17.6	30.2	48.0	69.6	84.8	92.5	80.4	46.9
2016–2017	9.7	10.1	12.2	17.5	30.3	48.0	69.6	85.0	92.7	80.5	47.3
2017–2018	9.2	9.8	12.0	17.8	30.6	48.3	69.5	85.1	92.8	80.6	47.7
2018–2019	8.8	9.9	12.3	17.7	30.7	48.4	69.3	84.8	92.4	80.4	47.3

Table 3 shows the age‐specific prevalence of all chronic disease groups studied for the 2018–2019 season. In this season, the prevalence of chronic circulatory diseases was 36.9% in the overall population, followed by chronic respiratory diseases (11.1%), diseases associated with a weakened immune system (10.7%), and diabetes (10.6%). Beginning with the age group of 30–39 years, circulatory diseases were the leading group of chronic diseases, mainly due to hypertension. In the age groups ≤30 years, chronic respiratory diseases were the group with the highest prevalence.

**TABLE 3:** Age‐specific proportion of population (%) with specific chronic conditions in the 2018–2019 season

	Age (years)										
Type of chronic condition	1–9	10–19	20–29	30–39	40–49	50–59	60–69	70–79	≥80	≥60	Total
Respiratory	5.83	6.88	6.37	6.68	8.75	10.77	14.10	16.51	17.13	15.68	11.07
Asthma	4.60	6.20	5.39	5.49	6.78	7.10	7.30	7.58	6.51	7.21	6.58
COPD	1.14	0.52	0.34	0.58	1.46	3.39	6.83	9.13	10.17	8.45	4.24
Circulatory	1.84	1.42	3.72	7.86	18.15	34.89	56.99	75.31	86.33	70.55	36.91
Hypertension	0.04	0.31	2.32	6.09	15.32	30.96	51.85	68.61	78.36	64.17	32.93
Heart failure	0.02	0.02	0.05	0.13	0.39	1.12	3.30	7.95	17.69	8.41	3.53
Ischemic heart diseases	0.00	0.00	0.02	0.12	0.68	2.66	7.81	16.17	25.51	15.03	6.39
Pulmonary heart diseases	0.02	0.01	0.05	0.11	0.21	0.38	0.82	1.89	3.11	1.75	0.79
Cerebrovascular diseases	0.05	0.05	0.12	0.25	0.76	2.32	6.44	13.88	20.71	12.51	5.39
Diabetes	0.13	0.43	0.71	1.44	3.53	7.49	16.45	24.73	28.48	22.28	10.61
Liver	0.03	0.11	0.53	1.42	3.33	6.18	10.25	12.22	10.63	11.04	5.95
Renal	0.03	0.05	0.17	0.35	0.82	1.79	4.61	10.91	19.89	10.52	4.55
Neurological	0.46	0.75	1.13	1.52	1.80	2.08	2.46	4.47	9.21	4.80	2.77
Multiple sclerosis	0.00	0.01	0.16	0.42	0.66	0.70	0.52	0.30	0.14	0.35	0.39
Immunocompromised	1.02	0.98	1.26	2.17	4.42	8.26	14.52	24.36	28.32	21.33	10.70
Cancer	0.11	0.16	0.36	0.83	2.04	4.47	8.67	14.99	16.86	12.88	6.15
HIV/AIDS	0.00	0.00	0.02	0.08	0.15	0.16	0.08	0.04	0.02	0.05	0.08
Disorders involving the immune mechanism	0.74	0.45	0.37	0.44	0.61	0.74	0.81	0.79	0.65	0.76	0.65

Abbreviations: AIDS, acquired immunodeficiency syndrome; COPD, chronic obstructive pulmonary disease; HIV, human immunodeficiency virus.

**FIGURE 2:**
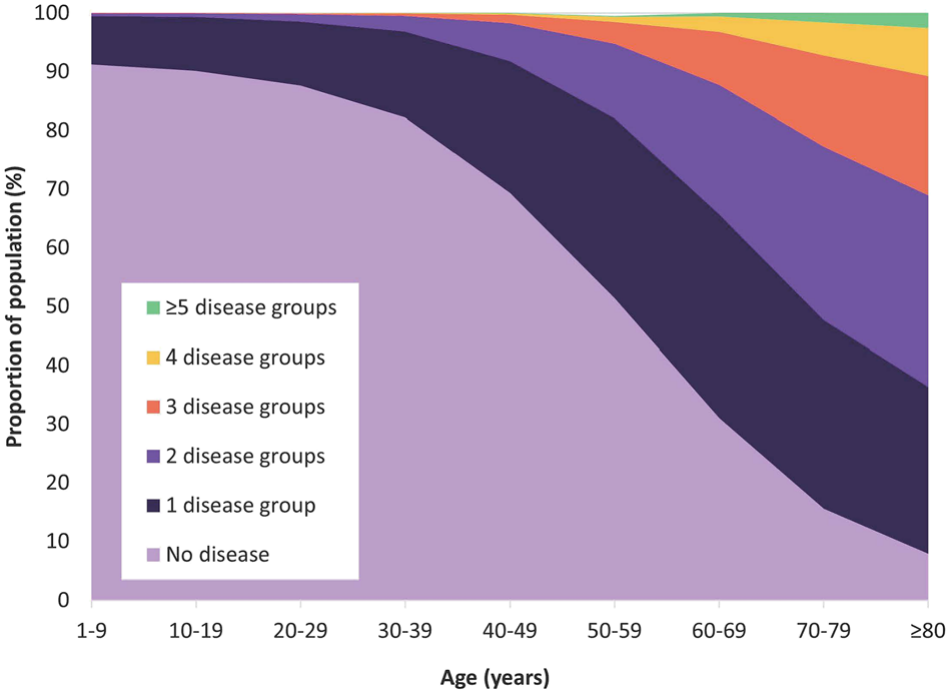
Proportion of individuals with underlying chronic conditions falling into the named disease groups (respiratory, circulatory, diabetes, liver, renal, neurological, immunocompromised) in the 2018–2019 season

Figure 2 provides an overview of the age‐specific morbidity profile in the 2018–2019 season. In the 50–59 years age group, one in two individuals already had at least one chronic condition. One‐third of the 60–69 years age group suffered from diseases from at least two of the disease groups studied. More details are given in the supporting information Table S2.

### 3.2 VCRs

**FIGURE 3:**
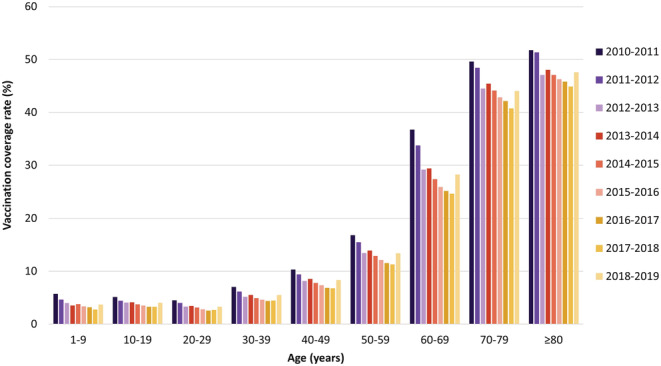
Age‐specific influenza vaccination coverage rates (%) in the 2010–2011 to 2018–2019 seasons

VCRs decreased over time in all age groups until the end of the 2017–2018 season (Figure 3). For example, in all individuals aged ≥60 years, the overall VCR decreased from 45% in the 2010–2011 season to 35% in the 2017–2018 season. A new increase in the VCRs in all age groups was observed in the 2018–2019 season (Table 4). Table 4 provides an overview of age‐specific VCRs in all the studied seasons. Influenza vaccine uptake varied widely with age. The highest VCRs were consistently found in people aged ≥60 years. Within this age group, rates were higher among those aged 70–79 years compared with those aged 60–69, while rates among those aged ≥80 were marginally higher than those aged 70–79. VCRs among females were slightly higher than among males in most age groups across all seasons.

**TABLE 4:** Age‐specific influenza vaccination coverage rates (%) in the 2010–2011 to 2018–2019 seasons, in the overall population and in individuals with or without chronic conditions

		Age (years)										
	Season	1–9	10–19	20–29	30–39	40–49	50–59	60–69	70–79	≥80	≥60	Total
Overall (no split by risk)	2010–2011	5.7	5.1	4.5	7.0	10.3	16.8	36.7	49.6	51.8	44.8	22.0
	2011–2012	4.6	4.4	4.0	6.1	9.4	15.5	33.7	48.4	51.3	43.2	20.9
	2012–2013	4.0	4.1	3.3	5.2	8.2	13.4	29.2	44.5	47.1	38.9	18.8
	2013–2014	3.5	4.1	3.4	5.5	8.6	13.9	29.4	45.4	48.0	39.6	19.3
	2014–2015	3.8	3.7	3.2	4.9	7.8	12.9	27.4	44.1	47.1	38.2	18.6
	2015–2016	3.4	3.5	2.8	4.6	7.4	12.1	25.9	42.9	46.3	36.9	18.2
	2016–2017	3.2	3.3	2.5	4.4	6.9	11.6	25.2	42.1	45.8	36.2	17.9
	2017–2018	2.8	3.3	2.7	4.4	6.8	11.3	24.7	40.8	44.9	35.2	17.7
	2018–2019	3.7	4.0	3.3	5.5	8.3	13.4	28.2	44.0	47.6	38.5	19.6
With chronic conditions	2010–2011	13.7	13.7	9.7	13.8	18.3	24.7	43.8	53.8	54.2	50.3	39.4
	2011–2012	12.0	12.7	8.8	12.3	16.7	22.9	40.6	52.6	53.7	48.7	37.8
	2012–2013	10.8	12.3	7.8	10.5	14.6	20.0	35.4	48.3	49.2	44.0	34.0
	2013–2014	9.8	12.2	7.8	11.1	15.0	20.5	35.6	49.3	50.2	44.8	34.6
	2014–2015	10.9	11.8	7.3	10.1	13.8	19.2	33.2	47.9	49.1	43.2	33.3
	2015–2016	10.0	11.4	6.7	9.4	12.9	18.0	31.5	46.5	48.2	41.7	32.2
	2016–2017	10.0	10.8	6.1	9.0	12.2	17.2	30.6	45.6	47.7	41.0	31.6
	2017–2018	9.4	11.1	6.5	9.0	11.9	16.7	29.9	44.1	46.7	39.9	31.0
	2018–2019	11.8	12.6	7.6	10.4	14.0	19.3	33.8	47.6	49.5	43.3	34.0
Without chronic conditions	2010–2011	4.7	4.1	3.9	5.7	7.5	10.3	21.7	30.1	28.9	25.0	8.8
	2011–2012	3.7	3.5	3.4	4.9	6.7	9.2	19.1	28.3	27.4	22.8	7.8
	2012–2013	3.1	3.2	2.8	4.1	5.6	7.7	15.8	25.4	24.3	19.5	6.7
	2013–2014	2.8	3.2	2.9	4.4	5.9	8.0	15.9	25.7	24.7	19.7	6.8
	2014–2015	2.9	2.8	2.6	3.8	5.3	7.2	14.4	24.3	23.8	18.3	6.2
	2015–2016	2.6	2.6	2.3	3.6	4.9	6.7	13.3	22.9	22.7	17.0	5.8
	2016–2017	2.5	2.4	2.0	3.4	4.6	6.4	12.7	22.2	22.7	16.3	5.5
	2017–2018	2.1	2.4	2.2	3.5	4.5	6.3	12.7	21.5	22.0	16.0	5.5
	2018–2019	2.9	3.1	2.7	4.5	5.8	7.9	15.6	24.3	23.8	18.8	6.8

**FIGURE 4:**
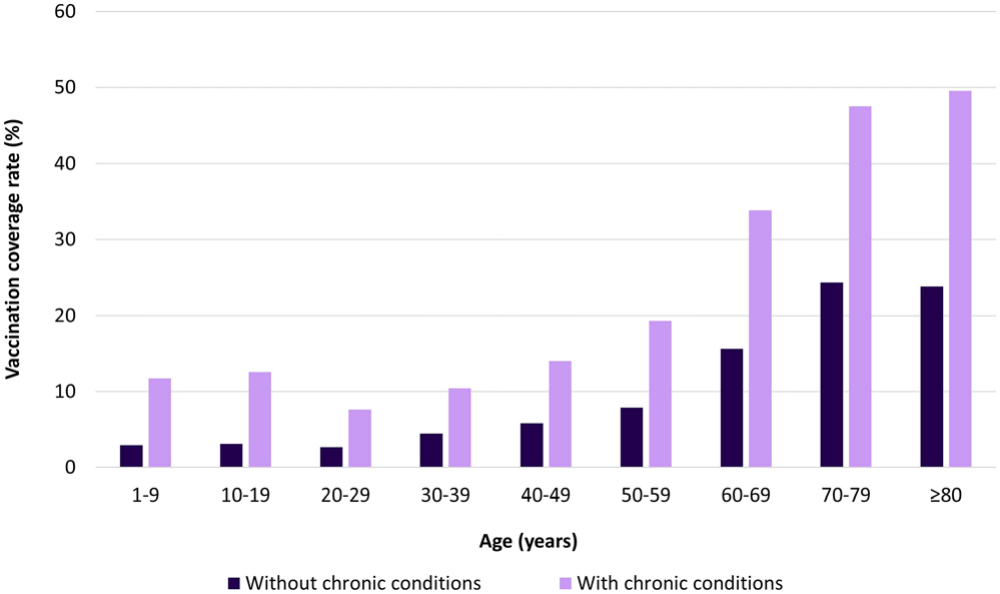
Age‐specific influenza vaccination coverage rates (%) in the population with and without chronic conditions in the 2018–2019 season

Coverage rates also varied between individuals with and without chronic conditions. In all age groups, influenza VCRs in individuals with underlying chronic conditions were higher than in those who were not chronically ill. In the 2018–2019 season, age‐specific coverage in individuals with chronic conditions ranged from 7.6% to 49.5%, and coverage in the healthy population ranged from 2.7% to 24.3% (Figure 4). In 2018–2019, the highest VCR of 49.5% was seen in individuals aged ≥80 years with chronic conditions. The rate in individuals aged ≥60 years with chronic conditions (a group that could be considered doubly at risk) was only 43.3%.

VCRs also varied with the type of chronic disease (Table 5). For example, vaccine uptake was lower in individuals with asthma than in those with ischemic heart disease, heart failure, or diabetes when looking at the coverage rates in the total group.

**TABLE 5:** Age‐specific influenza vaccination coverage rates (%) in specific at‐risk populations in the 2018–2019 season

	Age (years)										
Type of chronic condition	1–9	10–19	20–29	30–39	40–49	50–59	60–69	70–79	≥80	≥60	Total
Respiratory	12.3	12.8	6.7	10.2	15.1	22.6	38.7	52.0	53.6	47.6	33.4
Asthma	12.7	13.1	6.7	10.3	15.5	23.1	40.0	54.0	55.6	48.6	29.7
COPD	11.5	14.1	6.4	12.0	16.9	25.6	40.1	52.5	54.2	48.9	42.6
Circulatory	11.8	14.4	8.2	10.6	14.3	19.7	34.6	48.3	49.9	44.3	37.3
Hypertension	15.6	11.8	7.3	10.4	14.2	19.8	34.8	48.6	50.4	44.6	38.0
Heart failure	40.0	36.5	22.0	18.9	22.5	27.7	40.5	50.2	50.0	48.6	46.9
Ischemic heart diseases	36.4	26.1	11.6	17.0	19.5	26.4	40.5	52.7	53.6	50.5	48.3
Pulmonary heart diseases	39.7	37.8	15.8	15.5	20.7	27.4	42.0	52.3	53.2	50.7	47.1
Cerebrovascular diseases	15.4	16.0	12.2	13.7	16.3	22.2	36.7	49.3	50.8	47.3	44.6
Diabetes	18.6	17.8	10.8	13.9	17.9	25.4	39.3	50.8	52.2	47.8	43.1
Liver	20.5	10.1	8.6	10.2	14.4	21.0	35.6	48.7	50.7	44.2	36.9
Renal	23.5	24.0	14.9	19.4	22.0	27.6	41.3	52.3	52.6	50.5	47.9
Neurological	11.0	11.3	13.1	15.1	18.6	23.1	36.0	47.4	49.9	46.2	37.2
Multiple sclerosis	0.0	15.0	9.6	12.4	17.9	22.5	34.6	42.8	43.5	37.9	25.4
Immunocompromised	12.2	12.3	10.6	13.7	17.5	23.1	37.1	49.8	51.5	46.8	40.7
Cancer	12.8	10.3	9.4	10.8	15.5	21.6	36.1	48.9	50.8	46.0	41.0
HIV/AIDS	25.0	25.0	28.9	39.7	40.5	48.3	55.0	60.6	55.8	56.7	47.1
Disorders involving the immune mechanism	11.2	11.7	8.5	12.5	18.0	25.6	40.3	52.5	53.8	47.5	31.3

Abbreviations: AIDS, acquired immunodeficiency syndrome; COPD, chronic obstructive pulmonary disease; HIV, human immunodeficiency virus.

Influenza VCRs varied widely by region (Table 6), with the lowest coverage rates observed in the south of Germany (Baden‐Wuerttemberg and Bavaria). The highest VCRs were achieved in the eastern part of Germany (Berlin, Brandenburg, Mecklenburg–Western Pomerania, Saxony, Saxony‐Anhalt, and Thuringia).

**TABLE 6:** Regional influenza vaccination coverage rates (%) in individuals 60 years and older in the 2014–2015 to 2018–2019 seasons

	Season				
ASHIP region	2014–2015	2015–2016	2016–2017	2017–2018	2018–2019
Baden‐Wuerttemberg	25.7	24.7	24.5	24.6	26.1
Bavaria	24.4	23.9	23.8	23.8	26.5
Berlin	47.9	46.8	46.6	Not available	Not available
Brandenburg	56.8	55.0	54.3	54.2	56.2
Bremen	32.6	31.3	31.9	31.0	34.4
Hamburg	37.4	34.6	33.2	32.0	33.9
Hesse	35.3	34.3	33.1	31.9	33.6
Mecklenburg–Western Pomerania	49.3	50.1	50.0	49.8	52.3
North Rhine	35.3	33.6	32.9	32.0	33.4
Lower Saxony	39.3	38.0	37.6	37.5	40.6
Rhineland‐Palatinate	35.4	33.8	33.0	32.5	35.1
Saarland	31.9	31.0	30.2	29.8	33.7
Saxony	55.9	54.7	53.4	53.2	53.1
Saxony‐Anhalt	58.1	56.7	55.8	56.9	58.6
Schleswig‐Holstein	38.5	37.2	37.1	35.9	39.6
Thuringia	48.8	47.4	46.4	47.4	49.3
Westphalia‐Lippe	34.8	33.0	31.5	30.2	32.1

Abbreviation: ASHIP, Association of Statutory Health Insurance Physicians.

In the 2018–2019 season, 93.3% of all vaccinations took place from September to November (September: 10.4%; October: 51.9%; November: 31.0%). December and January accounted for 4.5% and 1.4% of all vaccinations, respectively. Most influenza vaccinations were administered by general practitioners (GPs) (92.1%), followed by gynecologists (3.0%) and pediatricians (2.6%).

## 4 DISCUSSION

Our analysis of the BARMER statutory health insurance database in Germany showed that the prevalence of chronic diseases has increased slightly over time, but VCRs have remained consistently and substantially below the WHO target throughout the period analyzed.

Approximately 80% of the population aged ≥60 years had at least one chronic condition, as did nearly 50% of the population aged 50–59. The morbidity profile was dominated by circulatory diseases, particularly among the older age groups. The proportion of the population with more than one chronic condition also increased with age. Individuals within this category accounted for an increased proportion of morbidity among older age groups, particularly those aged ≥60 years.

The prevalence data presented in our study for specific chronic diseases (based on both inpatient and outpatient diagnoses) are similar to those reported in other studies, which all used outpatient claims data of the complete German SHI population. In our study, diabetes prevalence was 10.6% overall with a marked increase between the 40‐ to 49‐ and 50‐ to 59‐year age groups and then between each subsequent age decade. This is in line with results reported in another German claims data analysis, where the prevalence of diabetes was 9.8% in 2015.^11^ The prevalence of asthma observed in our study was 6.6%, compared with 5.7% in another claims data analysis in 2016.^12^ A 2020 analysis of claims data by Holstiege et al. found that 28.4% of individuals had at least one diagnosis with hypertension in 2018; this is comparable with the prevalence of 32.9% observed in our study.^13^ Heart failure prevalence in our study was similar to that in another claims data analysis in 2017 (3.5% vs. 3.4%, respectively).^14^

Internationally, multiple studies have looked at the prevalence of high‐risk conditions for influenza. The prevalence of high‐risk conditions in Germany that we observed was slightly lower than a US study based on data for 2006–2008 from the National Health Interview Survey and other national data systems, although our study covers a more recent and longer time period.^15^ In contrast, a UK study using data collected by the Royal College of General Practitioners in 2003 reported a lower prevalence of chronic cardiovascular disease, chronic respiratory disease, and diabetes compared with our study.^10^ Data from Scotland in 2007 on multimorbidity, covering >1.7 million individuals, showed that the prevalence of multimorbidity increased with age, with nearly two‐thirds of individuals ≥65 years of age having multiple morbidities.^16^

Overall, 34% of individuals with chronic conditions and 38.5% of individuals ≥65 of age were vaccinated against influenza in the 2018–2019 season. VCRs were highly dependent on age and risk status, with older age groups and those with chronic conditions being associated with an increased VCR. Cross‐age VCRs could vary more between some conditions than the corresponding age‐specific values (e.g., asthma versus chronic obstructive pulmonary disease [COPD]) due to the different age‐specific prevalence.

Other studies support the findings that current VCRs in Germany are well below the WHO and European Union (EU)‐defined targets. VCRs among the ≥60 years age group in our study were comparable with estimates from the RKI.^17^ This is also true for age‐specific VCRs in individuals with underlying chronic diseases. Our findings are consistent with recent evidence that VCRs in Germany are significantly below those of many other European countries and are even below the EU‐27 average rates.^18^

A recent study by Akmatov et al. analyzed nationwide SHI‐physician outpatient claims data covering 87% of the German population from 2009–2018 to assess influenza vaccination uptake among chronically ill individuals.^19^ Coverage was slightly higher among females than males, and notably higher among older age groups, as noted in our study. Although many of that study's findings are similar to ours, there were key differences in the methods used.^19^ Firstly, Akmatov et al. focused only on the proportion of the population with chronic illnesses, whereas we took a broader approach—assessing the influenza VCRs in individuals with and without chronic conditions. Furthermore, most of the results in the study by Akmatov et al. are available only for specific disease groups. An aggregated value for the group of individuals with at least one underlying chronic disease is only given for the 2017–2018 season. Also, as in some of the other studies described above, Akmatov et al. only included outpatient diagnoses in their study, while our study includes both outpatient and inpatient diagnoses.^19^

A decrease in vaccination coverage over time in all age groups was observed, followed by an increase in the most recently recorded season, although VCRs remain below the level observed at the beginning of the decade. The WHO goal of 75% coverage or higher among older individuals and individuals with chronic illnesses has not yet been achieved.

There is a noticeable trend in the timing of vaccinations, with the majority taking place in October, followed by a sharp decline after November. This is in line with expectations that the population is encouraged to be vaccinated before the start of the influenza season. The STIKO recommends that annual influenza vaccination should take place in autumn.^6^

Regional differences included lower VCRs in the south of Germany and higher VCRs in the east. Rieck et al. also reported in 2021 that vaccination coverage against influenza was significantly higher in Eastern than in Western Germany.^17^ Similar regional trends in VCRs have been reported previously for other types of vaccination. Rieck et al. reported that VCRs were higher in Eastern than in Western Germany for tetanus and diphtheria, by as much as 20 percentage points over the 2016–2020 period.^20^ It is well‐known that the willingness to vaccinate among physicians and the willingness to get vaccinated in the general population are higher in the eastern than in the western federal states due to historical reasons.^21^

A study by Arlt et al., using data for the 2013–2014 season from a questionnaire sent to GPs across 16 districts with the highest and lowest VCRs in Western and Eastern Germany, suggested that GPs' attitudes towards vaccination against influenza and regional VCRs are correlated.^22^ The study also showed that the attitude patterns of GPs' patients and colleagues appear to influence the GPs and should therefore be included in future interventions to increase influenza vaccine uptake at a regional level.^22^

Bӧdeker et al. found that the most common reasons for low influenza VCR in Germany are a general mistrust of vaccination along with the perception that influenza is not a dangerous disease, both of which were stated by more than 20% of interviewees.^23^ For example, approximately half of individuals interviewed, aged ≥60 years or with an underlying chronic disease, believed that influenza vaccination was a cause of influenza. Bӧdeker et al. recommend that future communication strategies be targeted to tackle these perceptions.^23^

The factors affecting vaccination behavior were explored and categorized into the “Four C model” by Betsch et al. in 2015, where non‐vaccination is defined as being a result of either complacency, convenience, a lack of confidence, or a utility calculation.^24^ These factors can affect VCRs by influencing individuals' perceived risk of the disease versus their perceived risk from vaccination. Individuals can be dissuaded from vaccination if their perceived risk from the vaccine is greater than their perceived risk of the disease.^24^ Betsch et al. suggest that interventions that aimed to improve VCR should be targeted to the Four C areas by focusing on communicating the risk of disease, removing barriers to vaccination, and correcting misconceptions.^24^

Potential strategies to increase VCRs have also recently been explored by Kassianos et al. (2021) and could be implemented in the future to bring VCRs in Germany closer to target. The authors identified factors for achieving a high VCR by analyzing data from four benchmark countries with high VCRs: Australia, Canada, the UK, and the United States.^25^ Broadly, the factors that bring about a successful vaccination program can be categorized into five main areas: health authority accountability, facilitated access to vaccination, healthcare professional accountability and engagement, awareness of the burden and severity of disease, and a strong belief in the benefits of vaccination.^25^

One element that has been repeatedly mentioned as a relevant factor is patient reminder interventions.^26^, ^27^ A systematic review found that patient reminder and recall systems can effectively improve VCRs.^28^ Digital immunization management systems can help integrate patient reminders and invitations into the daily workflow of primary care practices.^29^

The current study possesses several strengths that reinforce the importance of our results. Firstly, this is one of the first publications for Germany with data on both size of the population at risk and associated VCRs in all age groups. Secondly, the study covered a large database across nine complete influenza seasons. Finally, a granular approach was taken in the definition of patients with chronic conditions and the analysis based on that classification.

However, the study is not without limitations. Notably, only SHI data were included and not private insurance data. In addition, the data included here only cover a relatively small proportion of the total German population and may not be fully representative of the entire nation. Data from children ≤1 year old were not included in this study or in the Akmatov study. Data from the 2019–2020 and 2020–2021 seasons were not yet available and could not be included in the analysis. Also, coverage during these seasons may have been affected by the presence of COVID‐19 in Germany. An analysis of influenza VCRs in the 2020–2021 season showed that coverage in the ≥60 years age group significantly increased from 38.8% in the previous season to 47.3%.^20^ This increase in VCRs could be due to heightened awareness and preparedness as a result of the COVID‐19 pandemic. Although it was not possible to evaluate data from Berlin separately, these data were included in their entirety in the overall analysis.

In addition, the definition of risk groups presents a challenge; other studies do not always transparently document the ICD‐10 codes used. Therefore, we are unable to confirm if the same codes were used across studies, which may reduce the strength of our comparisons with other analyses. Some codes may also have been entered into the database incorrectly. For example, in Table 4, the prevalence of COPD in children aged 1–9 is shown as 1.14%. Some physicians may have used this code when no specific cause for respiratory disease could be identified, or they may have used “COPD” as an umbrella term for lung diseases of a chronic obstructive nature. Nonetheless, the data that we present here provide valuable insight into influenza VCRs among the at‐risk population and how they have changed over time.

## 5 CONCLUSION

Our claims data analysis revealed insufficient influenza VCRs in individuals who are at high risk of suffering severe complications from influenza infection (elderly individuals and individuals with chronic diseases) despite recommendations by STIKO. These results suggest that intensified public health efforts are necessary to reach the WHO vaccination coverage target of 75%.

## AUTHOR CONTRIBUTIONS

**Oliver Damm:** Conceptualization; funding acquisition; methodology. **Anya Krefft:** Conceptualization. **Rolf Kramer:** Conceptualization; methodology. **Julian Witte:** Conceptualization; formal analysis; methodology; project administration. **Manuel Batram:** Conceptualization; formal analysis; methodology; software. **Wolfgang Greiner:** Conceptualization; formal analysis; methodology; supervision.

## CONFLICT OF INTEREST

OD, AK, JA, and RK are employees of Sanofi Deutschland GmbH and may hold shares and/or stock options in the company; JW and MB own shares of Vandage GmbH, which received consulting fees and grants from AstraZeneca, AOK Rheinland/Hamburg, BARMER, DAK‐Gesundheit, German G‐BA, GSK, Sanofi, Sequirus, and Techniker Krankenkasse; JS received fees for consulting and/or lecture services from Bavarian Nordic, BioNTech, GSK, Janssen, MSD, Mylan, Pfizer, Sanofi, Seqirus, and Takeda; WG is a member of the scientific advisory boards of the health insurance companies BARMER, DAK‐Gesundheit and Techniker Krankenkasse.

## ETHICS STATEMENT AND INFORMED CONSENT

As the study did not gather patient or individual‐level data or involve any interventions, formal ethical approval was not sought, and informed consent was not applicable.

### PEER REVIEW

The peer review history for this article is available at https://publons.com/publon/10.1111/irv.13054.

## Supporting information


**Figure S1:** Schematic representation of the study period for one influenza season (2017–2018)
**Table S1:** Size of study population per season
**Table S2:** Proportion of individuals (%) with underlying chronic conditions falling into the named disease groups (respiratory, circulatory, diabetes, liver, renal, neurological, immunocompromised) in the 2018–2019 season

## Data Availability

The data are not publicly available due to data protection regulations of the data holder and national legislation.
