# Supplementary material for: Prevalence of chronic conditions and influenza vaccination coverage rates in Germany: Results of a health insurance claims data analysis
Source: Influenza Other Respir Viruses. 2022 Oct 1;17(1):e13054. doi: 10.1111/irv.13054 (PMC9835435; doi:10.1111/irv.13054)
Supplement: Supplementary file 1 — Figure S1: Schematic representation of the study period for one influenza season (2017–2018) Table S1: Size of study population per season Table S2: Proportion of individuals (%) with underlying chronic conditions falling into the named disease groups (respiratory, circulatory, diabetes, liver, renal, neurological, immunocompromised) in the 2018–2019 season [file IRV-17-e13054-s001.docx]

# Supplementary materials


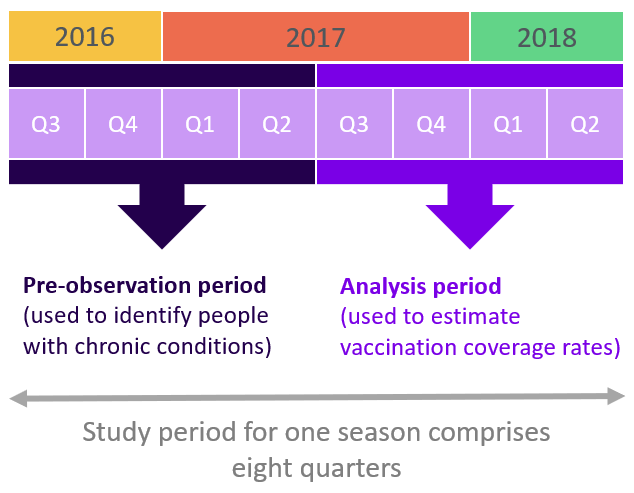


**Figure S1:** Schematic representation of the study period for one influenza season (2017–2018)

**Table S1:** Size of study population per season

| **Season** | **Age (years)** | | | | | | | | | |
| --- | --- | --- | --- | --- | --- | --- | --- | --- | --- | --- |
|  | **1–9** | **10–19** | **20–29** | **30–39** | **40–49** | **50–59** | **60–69** | **70–79** | **≥80** | **Total** |
| 2010–2011 | 397,695 | 535,704 | 371,946 | 460,632 | 846,079 | 869,272 | 765,596 | 765,847 | 360,350 | 5,373,121 |
| 2011–2012 | 416,158 | 581,534 | 437,695 | 545,362 | 929,321 | 992,625 | 854,154 | 881,079 | 415,484 | 6,053,412 |
| 2012–2013 | 435,840 | 622,797 | 520,835 | 664,504 | 1,026,916 | 1,143,888 | 984,560 | 999,487 | 489,593 | 6,888,420 |
| 2013–2014 | 446,484 | 621,707 | 537,086 | 694,846 | 1,002,660 | 1,191,947 | 1,004,882 | 1,042,301 | 496,016 | 7,037,929 |
| 2014–2015 | 436,104 | 602,289 | 531,739 | 709,417 | 959,645 | 1,222,229 | 1,016,018 | 1,061,432 | 519,696 | 7,058,569 |
| 2015–2016 | 422,903 | 556,261 | 516,795 | 707,113 | 899,663 | 1,217,078 | 1,034,441 | 1,034,774 | 549,017 | 6,938,045 |
| 2016–2017 | 423,785 | 532,218 | 504,112 | 710,357 | 851,879 | 1,216,205 | 1,055,681 | 1,007,515 | 582,319 | 6,884,071 |
| 2017–2018 | 398,641 | 502,117 | 469,075 | 666,695 | 770,716 | 1,156,028 | 1,021,703 | 934,701 | 583,748 | 6,503,424 |
| 2018–2019 | 418,933 | 516,616 | 516,823 | 712,922 | 768,332 | 1,178,057 | 1,054,920 | 923,432 | 628,032 | 6,718,067 |

**Table S2:** Proportion of individuals (%) with underlying chronic conditions falling into the named disease groups (respiratory, circulatory, diabetes, liver, renal, neurological, immunocompromised) in the 2018–2019 season

| **Number of chronic disease groups** | **Age (years)** | | | | | | | | |
| --- | --- | --- | --- | --- | --- | --- | --- | --- | --- |
|  | **1–9** | **10–19** | **20–29** | **30–39** | **40–49** | **50–59** | **60–69** | **70–79** | **≥80** |
| No disease | 91.19 | 90.10 | 87.63 | 82.24 | 69.39 | 51.33 | 31.01 | 15.54 | 7.86 |
| 1 | 8.31 | 9.23 | 10.90 | 14.60 | 22.44 | 30.73 | 34.70 | 32.13 | 28.53 |
| 2 | 0.47 | 0.62 | 1.27 | 2.64 | 6.42 | 12.67 | 22.03 | 29.54 | 32.58 |
| 3 | 0.02 | 0.04 | 0.13 | 0.44 | 1.45 | 3.77 | 9.02 | 15.52 | 20.31 |
| 4 | 0.00 | 0.00 | 0.02 | 0.07 | 0.26 | 0.85 | 2.64 | 5.67 | 8.16 |
| ≥5 | 0.00 | 0.00 | 0.00 | 0.01 | 0.04 | 0.16 | 0.60 | 1.60 | 2.55 |
